# Single-Cell Analysis of Circulating Tumor Cells from Patients with Colorectal Cancer Captured with a Dielectrophoresis-Based Micropore System

**DOI:** 10.3390/biomedicines11010203

**Published:** 2023-01-13

**Authors:** Masatoshi Nomura, Yuichiro Miyake, Akira Inoue, Yuhki Yokoyama, Nanaka Noda, Shihori Kouda, Tsuyoshi Hata, Takayuki Ogino, Norikatsu Miyoshi, Hidekazu Takahashi, Mamoru Uemura, Tsunekazu Mizushima, Yuichiro Doki, Hidetoshi Eguchi, Hirofumi Yamamoto

**Affiliations:** 1 Department of Gastroenterological Surgery, Graduate School of Medicine, Osaka University, Suita 565-0871, Japan; 2 Department of Gastroenterological Surgery, Sakai City Medical Center, Sakai 593-8304, Japan; 3 Department of Gastroenterological Surgery, Osaka General Medical Center, Osaka 558-8558, Japan; 4 Department of Molecular Pathology, Division of Health Sciences, Graduate School of Medicine, Osaka University, Suita 565-0871, Japan; 5 Department of Gastroenterological Surgery, Osaka Police Hospital, Osaka 543-0035, Japan

**Keywords:** circulating tumor cell, colorectal cancer, KRAS, BRAF, PIK3CA, single cell analysis, dielectrophoresis-based capture in micropore system

## Abstract

This study aimed to analyze circulating tumor cells (CTCs) from patients with colorectal cancer (CRC). We designed a dielectrophoresis-based micropore system and tested its cell capture with HT29 colon cancer cells. Then, blood samples were drawn from 24 patients with stages II-IV CRC. Mononuclear cells were isolated and loaded into the micropore system. Single cells were positioned into small pores with dielectrophoresis. After labeling the cells with the appropriate antibodies, tumor-like cells were collected with an automated micromanipulator. We collected 43 CTCs from 15 out of 24 patient samples. The presence of CTC was significantly associated with ling metastasis. We performed whole genome amplification, followed by PCR and Sanger sequencing, to examine the point mutations in the *KRAS*, *BRAF*, and *PIK3CA* genes. This mutation analysis was successfully performed in 35 cells. Among the 14 cytokeratin (CK)-positive cells, we found *PIK3CA* mutations in three cells (21%) from two patients. Among the 21 CK-negative cells, we found a *KRAS* mutation in one cell (5%) from one patient and a *PIK3CA* mutation in one cell (5%) from one patient. It is noteworthy that these mutations were not detected in the corresponding primary tumors. In conclusion, dielectrophoresis-based capture in a micropore system was useful for detecting both CK-positive and CK-negative CTCs. This simple method could be applied to various tumor types.

## 1. Introduction

Colorectal cancer (CRC) is the third most common cancer in the United States. In 2017, an estimated 1.4 million new CRC cases were diagnosed, and 0.5 million deaths were CRC-related [1]. It is worth noting that approximately 90% of cancer deaths are caused by distant metastasis [2]. A cancer diagnosis is typically determined by a histological examination of a tumor tissue biopsy. However, this examination provides only fragmentary information due to the heterogeneity of cells within a tumor. Furthermore, tumor tissue biopsies often require an invasive procedure, such as major surgery. In addition, biopsies are sometimes difficult to acquire, particularly when the tumor resides deep in the body. In recent years, the liquid biopsy methodology has attracted attention [3]. Remarkable progress in the next-generation sequencing technique has brought circulating tumor DNA (ctDNA) to the stage of clinical testing [4]. The mutational status of ctDNA in genes such as *EGFR* and *KRAS* and DNA microsatellite instability can be monitored in blood samples. This methodology is expected to be utilized in the early diagnosis of cancer, in monitoring therapeutic efficacy, and in predicting cancer relapse [3,4,5,6,7].

Circulating tumor cells (CTCs) are another analyte available in liquid biopsies. Tumor cells with an invasive ability are released from the primary lesions into the blood, and some possess metastatic ability. The difference between ctDNA and CTCs is that the entire cell can be analyzed with CTCs, in addition to the tumor-derived DNA. To detect CTCs, an epithelial marker, EpCAM, is often used in Cell Search systems [8]. Monitoring the number of CTCs allows for predictions of relapse and metastasis, evaluations of minimal residual disease, and measurements of treatment response in patients with cancer [9,10]. It is known that the number of CTCs is significantly correlated with the prognosis in carcinomas of the colorectum, prostate, and breast [11,12,13].

Precise, sensitive methods for capturing CTCs are under development for use in clinical practice. Several methods can be used to collect CTCs, based on the physical properties of tumor cells. These include a single spiral microchannel, Ficoll-hypaque density-gradient centrifugation, microfiltration, and microfluidics [14]. However, the paucity of CTCs in whole blood is a major challenge in this research. It is reported that the number of CTCs is typically less than five in 10 mL of peripheral blood in patients with CRC [15]. Thus, the loss of CTCs during the enrichment and isolation processes poses a serious problem. Nevertheless, the excessive circulation of CTCs allows contamination of a large number of blood cells. 

This study aimed to devise and test a dielectrophoretic micropore system for efficiently capturing CTCs from patients with CRC. All of the cells were subjected to immunostaining with anti-CD45 and anti-cytokeratin (CK) antibodies within the pores. Next, tumor-like cells were collected with an automated micromanipulator apparatus. To ensure the captured cells were truly tumor cells, we performed single cell analyses, which included whole-genome amplification, followed by the detection of mutations in the *KRAS*, *BRAF*, and *PIK3CA* genes, which are alterations representative of CRC [16]. For this purpose, we first established the technique with commercial CRC cell lines; then, we applied the method to clinical CTC samples that were obtained from 24 CRC patients. Our data suggested that this method was simple and useful for capturing and examining CTCs, even in CRC blood samples, where CTCs are relatively rare. 

## 2. Materials and Methods

### 2.1. Cell Culture 

The CRC cell lines, HT29, HCT116, DLD-1, and RKO, were obtained from the American Type Culture Collection (ATCC). The cells were cultured in Dulbecco’s modified Eagle’s medium (DMEM) supplemented with 10% fetal bovine serum (FBS), penicillin and streptomycin (100 units/mL, 100 µg/mL). The cells were incubated at 37 °C in a humidified atmosphere containing 5% CO_2_.

### 2.2. Spike Test with a Colon Cancer Cell Line

Cells from a colon cancer (HT29) cell line were mixed into venous peripheral blood from healthy donors. After centrifugation in a Ficoll solution, the isolated mononuclear cells were transferred to the microwell array and positioned into the pores with dielectrophoresis. Then, the cells were immunostained with anti-cytokeratin (CK) and anti-CD45 antibodies, and the number of CK^+^ CD45^−^ cells was counted.

### 2.3. Buffer, Reagents, and Antibodies 

A lysing solution, containing 9.0 g/L NH_4_Cl, 1.0 g/L KHCO_3_, and 0.037 g/L EDTA-4Na, was used to lyse the red blood cells. A 300-mM mannitol solution (Sigma Aldrich, St. Louis, MO, USA) was used for dielectrophoresis because it had suitable conductivity and appropriate osmotic pressure to allow living cells to maintain their shape. The surfaces of the microwell arrays were pre-coated with 0.1% bovine serum albumin (BSA; Sigma Aldrich, St. Louis, MO, USA) in phosphate-buffered saline (PBS). Distilled water containing 0.01% poly-L-lysine was used for the cell attachment procedure. The chamber-washing solution consisted of PBS with 1% BSA and 0.05% polyoxyethylene sorbitan monolaurate (Wako Pure Chemical Industries, Kyoto, Japan).

A fluorescein isothiocyanate (FITC)-conjugated anti-CK monoclonal antibody (mAb), CK3-6H5 (mouse IgG_1_), a phycoerythrin (PE)-conjugated anti-CD45 mAb, 5B1 (mouse IgG_2a_), and an FcR-blocking reagent were purchased from Milenyi Biotec (Bergisch-Gladbach, Germany). An Alexa Fluor 488-conjugated anti-Pan-CK mAb, AE1/AE3 (mouse IgG_1_), was purchased from Thermo Fisher Scientific (Santa Clara, CA, USA). A 4′,6-Diamidino-2-phenylindole dihydrochloride (DAPI) solution was purchased from DOJINDO Laboratories (Kumamoto, Japan).

### 2.4. Enrichment of Blood Samples 

**Figure 1:**
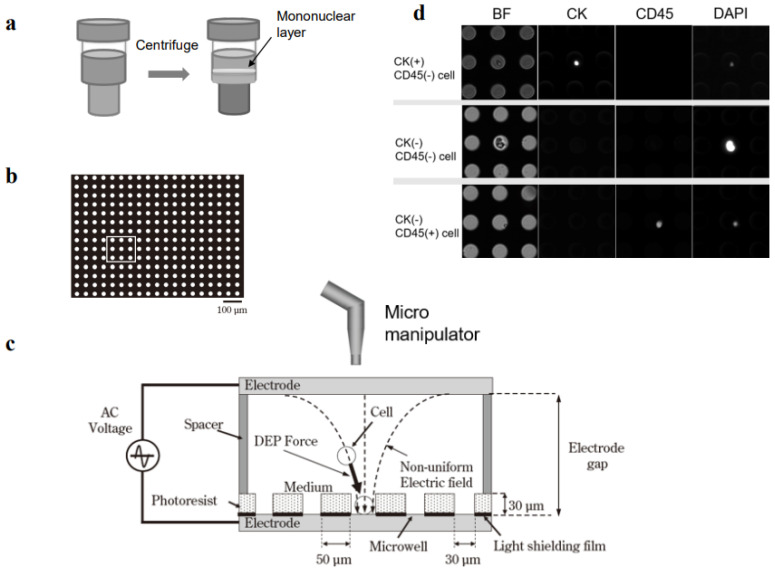
Single-cell collection in a dielectrophoretic micropore system. (**a**) Mononuclear cells are collected from 3 mL of peripheral blood with Ficoll density-gradient centrifugation. (**b**) Mononuclear cells are fixed in formalin and membranes are permeabilized. (**c**) Dielectrophoresis is carried out to position the cells into the pores (microwells) in the stage. Target single cells are collected with an automated micromanipulator. (**d**) Mononuclear cells are immunostained with antibodies specific for cytokeratin (CK) and CD45.

All of the blood samples were collected before surgery, or before newly scheduled chemotherapy. Three mL of peripheral venous blood was collected and transferred to a 5 mL blood collection tube containing EDTA (TERUMO, Tokyo, Japan). In a Tosoh enrichment tube (Figure 1a), 3.5 mL Ficoll (GE Healthcare, Chicago, IL, USA), 3 mL blood sample, 3 mL fixative solution, and 180 μL Tirofiban were added and mixed well. After standing for 15 min at room temperature (RT), 75 μL of RosetteSep reagent Human CD45 Depletion Cocktail (STEMCELL technologies, Vancouver, BC, Canada) was added to deplete the white blood cells and the red blood cells. After centrifugation (2000× *g*, 10 min, 24 °C), the concentrated mononuclear layer was collected, mixed with a lysing solution, then centrifuged at 300× *g* for 10 min at 24 °C. After removing the supernatant, the pellet was resuspended in 30 mL of polyethylene glycol bovine serum albumin (PEG-BSA) and centrifuged at 300× *g* for 5 min at 24 °C. This procedure was repeated three times. After removing the supernatant, the final pellet was suspended in PEG-BSA.

### 2.5. Tumor Cell Entrapment with Dielectrophoresis

The cell entrapment chamber was filled with Tween-20 in 300 mM mannitol and the cell suspension was injected through the injection port. The cells were positioned in the microwell array with dielectrophoresis (Figure 1b,c). The theoretical mechanism of dielectrophoresis has been described previously [17]. Briefly, a suspension of tumor cell-enriched mononucleated cells was loaded into the cell entrapment chamber of the micropore system. Then, a function generator (WF1974, NF Corporation, Tokyo, Japan) was immediately applied to generate 20 Vp-p AC voltage, with a square-wave shape, at a frequency of 3 MHz, for 3 min. This dielectrophoretic force moved the cells until they were entrapped in the microwells.

### 2.6. Immunofluorescence Staining

A poly-L-lysine solution (0.01%) was introduced into the chamber with continuously applied dielectrophoretic force. Next, the cells were incubated for 3 min at RT to allow cell attachment to the bottom of the wells. After removing the poly-L-lysine solution, 80% ethanol was added to the chamber, and the cells were incubated for 10 min at RT to allow permeabilization and fixation. After removing the ethanol solution, the chamber was washed with PBS containing 0.05% Tween-20 four times; then, FcR-blocking buffer with Tween-20 and 1% BSA in PBS was added for 10 min at RT. Subsequently, the chamber was filled with an immunofluorescent staining reagent, which included the CK-FITC antibody (Milenyi Biotec), Alexa Fluor 488-conjugated anti-Pan-CK mAb AE1/AE3 (Thermo Fisher Scientific), CD45-PE antibody (Milenyi Biotec), and DAPI in PBS with 0.05% Tween-20 and 1% BSA for 30 min. The chamber was then washed with PBS containing 0.05% Tween-20, and the images were examined with a fluorescence microscope. Images of the entire micropore array were acquired with an ADT-100 EM-CCD camera (FLOVEL, Tokyo, Japan), at the appropriate fluorescence wavelengths for DAPI, FITC, and PE. In addition, bright field images were acquired. 

### 2.7. Mutation Analysis

#### 2.7.1. Whole Genome Amplification from a Single Cell

Each single cell was stored in 30 mL sterile distilled water in 600-mL tube at −80 °C. Then, the single cells were thawed, and the whole genome of each cell was amplified with an Ampli1 WGA Kit (Menarini Silicon Biosystem, Bologna, Italy). 

#### 2.7.2. Tissue DNA

DNA was extracted from the primary CRC tumor tissues fixed in paraffin blocks. The extractions were performed with a QIAamp DNA FFPE Tissue Kit (QIAGEN, Tokyo, Japan). 

#### 2.7.3. Polymerase Chain Reaction and Sanger Sequencing

Polymerase chain reaction (PCR) primers were designed with primer-BLAST for the following mutational hotspots: *KRAS*, exon 2 codons 12 and 13; *BRAF*, exon 15 codon 600; and *PIK3CA*, exon 9 codon 1047. The primer sequences were:

*KRAS*  F: AGGCCTGCTGAAAATGACTGA,
  R: TGTTGGATCATATTCGTCCACAA
*BRAF*  F: TGCTTGCTCTGATAGGAAAATGAG,
  R: GGACCCACTCCATCGAGATT
*PIK3CA*  F: TGGAAACTTGCACCCTGTTT,
  R: TTGTCCATCGTCTTTCACCA

DNA amplifications, including the point mutation regions in *KRAS*, *BRAF*, and *PIK3CA*, were performed with PCR and the Ampli Taq Gold kit (Thermo Fisher Scientific). The PCR products were confirmed by electrophoresis. The bands that migrated at the correct molecular weight were purified with the Min Elute PCR Purification Kit (QIAGEN). Sanger sequencing was performed with the Big Dye terminator v3.1 Cycle sequencing Kit (Thermo Fisher Scientific). 

### 2.8. Patients

Between February 2015 and July 2016, blood samples were collected from 24 patients with CRC. Of these, 12 had synchronous metastasis at stage IV, and 9 had metachronous metastasis. The staging was determined according to the 8th version of the Union for International Cancer Control TNM classification for CRC [18]. Twelve patients received chemotherapy before the blood sample collection. The chemotherapy regimens included capecitabine, capecitabine and oxaliplatin (XELOX), uracil-tegafur (UFT) and leucovorin (UFT/LV), 5-fluorouracil (5-FU) and oxaliplatin (FOLFOX), 5-FU and irinotecan (FOLFIRI), titanium silicate-1 and oxaliplatin and bevacizumab (SOX+Bev), XELOX and bevacizumab (XELOX+Bev), irinotecan and panitumumab (CPT11+Pmab), FOLFOX and bevacizumab (FOLFOX+Bev), or FOLFIRI and bevacizumab (FOLFIRI+Bev) (Supplementary Table S1). Informed consent was obtained from all participants included in this study. This study was approved by the Institutional Review Board (Approval number 14,070 and 661-3). 

### 2.9. Clinical and Pathological Correlation

CRC cases with and without CTC were examined with regard to age, gender, serum CEA and CA19-9 levels, the location of the tumor, tumor size, the differentiation degree, stage, metastasis type (metachronous or synchronous), history of chemotherapy, presence of liver metastasis, presence of lung metastasis. 

### 2.10. Statistical Analysis

All of the data are expressed as mean ± standard deviation, or the median and interquartile range (IQR). Statistical differences were analyzed using Student’s t test for continuous variables and the Chi-squared test for non-continuous data. All of the statistical analyses were conducted with JMP ver. 14.0 (SAS Institute, Inc., Cary, NC, USA). A *p* value < 0.05 was considered to indicate statistical significance.

## 3. Results

### 3.1. Spike Test

HT29 cells were mixed into healthy donor venous peripheral blood at densities of 0, 12, 23, and 72 cells, in 3 mL blood. After centrifugation in Ficoll solution, the mononuclear cells were transferred to the microwell array and positioned into the pores with dielectrophoresis. Then, the cells were immunostained with anti-CK and anti-CD45 antibodies, and the numbers of CK^+^/CD45^−^ cells were counted (Figure 1a–d). The recovery rate was calculated as the number of cells recovered divided by the number of cells spiked (×100%). The cell recovery rates ranged between 75.0 and 78.2% (Supplementary Figure S1). 

### 3.2. Single-Cell Analysis of Mutations in KRAS, BRAF, and PIK3CA in CRC Cell Lines

**Figure 2:**
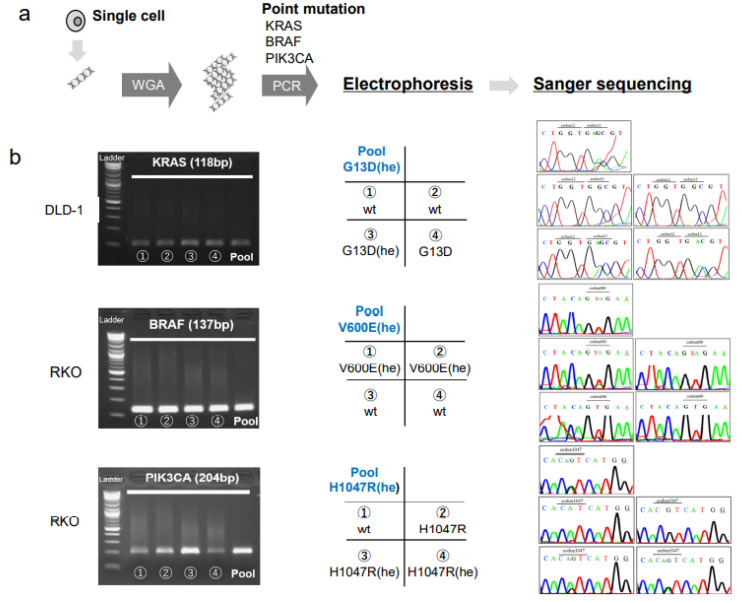
Single cell analyses of mutations in colorectal cancer cell lines. (**a**) DNA isolated from each single cell is subjected to whole genome amplification (WGA), followed by PCR at the hotspot mutation regions and Sanger sequencing. (**b**, *left column*) Electrophoresis of PCR products show specific amplifications of the *KRAS* gene in DLD-1 cells, the *BRAF* gene in RKO cells, and the *PIK3CA* gene in RKO cells. (*Middle column*) Matrices show the arrangement of data in the right column; Pool: pooled cells; cell numbers (1–4) and the genotypes indicated correspond to the single-cell PCR products shown in the left column; (*Right column*) Sanger sequencing results for each sample, arranged as shown in the middle column. The mutated codons are indicated at the top of each panel. Abbreviations: he: heterozygous, wt: wild type.

To detect point mutations within the hotspot regions of the *KRAS*, *BRAF*, and *PIK3CA* genes, the DNA content from a single cell from a CRC cell line (DLD-1, RKO, or HCT116) was subjected to whole genome amplification (WGA), followed by PCR and Sanger sequencing (Figure 2a). In addition to the single CRC cells, the pooled CRC cells were also analyzed. The primer melting temperatures (Tm) were tested from 55 °C to 64 °C for each primer, with the DNA extracted from the CRC cell lines. After the validation of the PCR products, the Tm was determined to be 59 °C (Supplementary Figure S2).

We found that the pooled cells of the DLD-1, RKO, and HCT116 cell lines had mutations in one or two genes, among *KRAS*, *BRAF*, and *PIK3CA*, consistent with previous studies [16]. We found a G13D (he) mutation in *KRAS*, the V600E (he) mutation in *BRAF*, and the H1047R (he) mutation in *PIK3CA* (Figure 2). On the other hand, the single cell analysis revealed a heterogenous gene status for *KRAS* in the DLD-1 cells, and for *BRAF* and *PIK3CA* in the RKO cells (Figure 2, Table 1). Heterogeneity in the mutation status among single cells was also found for the *KRAS* and *PIK3CA* genes in HCT116 cells (Table 1).

### 3.3. Single-Cell Analysis in CTCs from Patients with CRC 

Blood samples were collected from 24 patients with CRC. Of these, 21 patients had synchronous or metachronous metastases in various organs. However, metastases were not found in one patient with stage II CRC (Patient No. 23) and two patients with stage III CRC (Patient Nos. 2 and 14; Table 2).

A total of 43 cells, including both CK^+^/CD45^−^ and CK^−^/CD45^−^, were collected from 15 patient samples with the automated micromanipulator (Figure 1c). CK^+^/CD45^−^ cells were found in seven patients (Patient numbers [Pt. Nos.] 5–8, 12, 15, and 17), at cell counts of between one and five (Table 2). These patients had distant metastases to the liver, lung, and bone or recurrence in the peritoneum. In addition, nine patients (Pt. Nos.16–24), despite the CK^−^CD45^−^ status, had CTC-like cells, based on morphological features (i.e., enlarged, irregularly shaped nucleus; Table 2). Of these, one patient (Pt. No.17) had both CK^+^ and CK^−^ CTC-like cells. 

**Figure 3:**
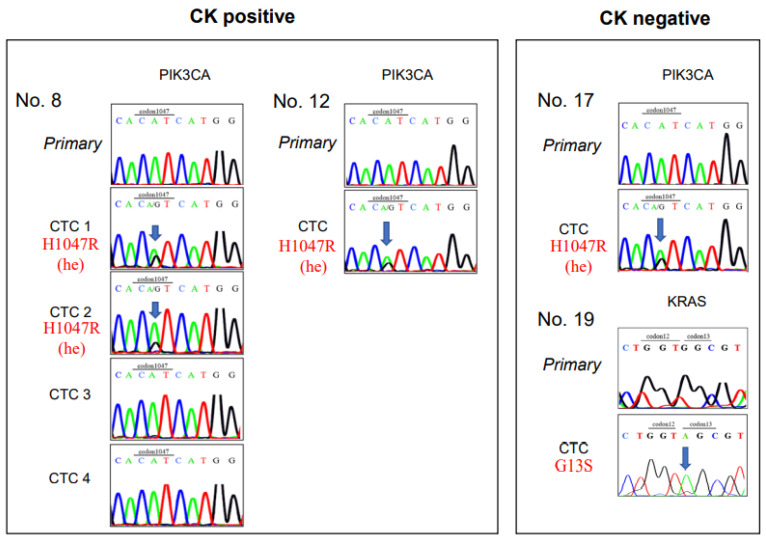
**Sanger sequencing of CK-positive CTCs and CK-negative CTCs.** Sanger sequences show gene mutations in clinical samples from patients with colorectal cancer. (**Left**) Sequences are shown for five CK-positive CTCs, including 4 CTCs from Patient number 8 (Pt. No. 8) and one CTC from Pt. No. 12. (**Right**) Sequences are shown for two CK-negative CTCs, including one each from Pt. Nos. 17 and 19. Primary: cell from the primary tumor; CK: cytokeratin; CTC: circulating tumor cell.

Mutation analysis was successfully performed in 35 of the 43 collected cells (Table 3) and in 14 of 15 patient samples (all except Pt. No. 21). Among the 14 CK^+^/CD45^−^ CTC candidates, the *PIK3CA* mutation was found in three cells (Pt. Nos. 8 and 12); however, in these cells, the *KRAS* and *BRAF* mutations were not detected (Figure 3). On the other hand, among the 21 CK^−^/CD45^−^ CTC-like cells, one cell showed a *KRAS* mutation (Pt. No. 19), and another cell displayed a *PIK3CA* mutation (Pt. No. 17), but none showed the *BRAF* mutation (Figure 3, Table 3).

### 3.4. Comparison of Mutations between CTCs and Primary Tumors

Paraffin-embedded blocks of the primary CRC samples were available from 12 of the 14 patients with CRC whose CTCs were examined in the mutation analysis. Two CRC tissue samples had *KRAS* gene mutations (15.4%, Pt. Nos. 7 and 12), but *KRAS* mutations were not detected in the CTCs of these two patients (Table 3). In contrast, in one of four CTCs in Pt. No. 19 showed a *KRAS* mutation, but the primary CRC sample had the wild type *KRAS* gene status. In addition, *PIK3CA* gene mutations were observed in the CTCs from Pt. Nos. 8, 12, 17, but their primary CRC tumors displayed the wild type *PIK3CA* gene (Table 3).

### 3.5. Relationship between CTC and Clinical and Pathological Parameters

CTC-positive cases and CTC-negative cases were compared with the various clinical and pathological parameters listed in Table 4. Among them, the presence of lung metastasis alone was significantly associated with the presence of CTC (*p* = 0.02).

## 4. Discussion

Although various systems have been developed in attempts to capture CTCs in blood samples from patients with cancer [8,18,19,20], a common problem is the loss of rare CTCs during the enrichment and isolation steps [8,21,22]. Studies have shown that a majority of patients with CRC (75%) had less than ten CTCs in a 10-mL blood sample [15]. This low number contrasts with findings in breast and prostate cancers, where CTCs are frequently detectable [23]. Our approach was to set up a dielectrophoretic system combined with a small stage, with an array of numerous pores. This system had several advantages, but, in particular, it allowed us to collect single, tumor-like cells directly, with an automated micromanipulator. On the other hand, a potential disadvantage in our system was that its capacity was small (3-mL blood samples) for each examination due to the limited number of pores (300,000) on the stage. Therefore, two rounds of examination might be necessary to obtain more CTCs. To secure rare CTCs, it is essential to prevent the loss of CTCs. Our spike test was well tolerated; thus, the recovery rate for the HT29 CRC cells was approximately 75%, which was comparable or better than the rates achieved with other systems [18,24,25].

To identify CTC-like cells, we detected CK expression with immunostaining in the first consecutive 15 samples. We previously reported that the pan-CK antibody, AE1/AE3, showed a nearly 100% coverage rate in the CRC samples [26]. In addition, cells with epithelial features were detected when we performed fluorescence immunostaining to distinguish CK ^+^/CD45^−^ cells. However, studies have also shown that the cells undergoing epithelial-mesenchymal transition lose the expression of the epithelial markers CK and EpCAM. Instead, those cells induce the expression of the mesenchymal marker, plastin 3, or cell surface vimentin (CSV) in CTCs [23,27]. It has also been suggested that those CTCs are more capable of adapting to a secondary site and, thus, they form distant metastases [28,29,30]. Indeed, based on the cytological appearances, we were aware of the presence of tumor-like cells that lacked CK expression. They had enlarged, irregularly shaped nuclei and, thus, an increased ratio of nucleus to cytoplasm. Moreover, we also found cells that exhibited pathological, tumor-like morphology; these cells comprised both epithelial and non-epithelial CTCs. Among these (e.g., patients 16–24), some were indeed cancer cells that displayed point mutations in the *KRAS* and *PIK3CA* genes (Pt. Nos.17 and 19). These findings indicated that our micropore system could facilitate the capture of CTCs with epithelial or mesenchymal features. Furthermore, triple staining for CK/CSV/CD45 may facilitate a more efficient detection of CTCs. Overall, this study showed that our micropore system could isolate CTC-like cells in 15 of 24 CRC samples (Table 2).

The greatest merit of this method was the automated micromanipulator, which facilitated single cell analysis by enabling the collection of single cells. To verify whether CTC-like cells were truly cancer cells, we performed single-cell mutation analyses of the *KRAS*, *BRAF*, and *PIK3CA* genes. Each gene has a hotspot of point mutations; thus, the presence of mutations could be readily evaluated with PCR by targeting the hotspot region and by performing Sanger sequencing. It was reported that the mutation rates in CRC were 37.6% in *KRAS*, 11.5% in *BRAF*, and 19.2% in *PIK3CA* [31,32].

To validate our method, we first worked with the CRC cell lines and performed a WGA. Then, we PCR-amplified the hotspot regions and performed the Sanger sequencing to identify those containing one base mutation. It is known that amplifying a WGA-based sequence with PCR can detect wild type genes and homozygous mutations correctly, but the rate of identifying heterozygous mutations is 25–75%. This limitation is due to the unequal amplification after WGA, which results in the loss of one allele (termed allele dropout: ADO) [33]. Consistently, our data of the CRC cell lines showed that the rate of detecting mutations was 25–75% (including heterozygous genes and ADOs of the wild type allele) in *KRAS*, *BRAF*, and *PIK3CA* (Table 1, highlighted in grey). In addition, in two of our clinical samples (from Pt. Nos. 7 and 12), the *KRAS* mutations in primary tumors were not detected in CTCs (Table 3, highlighted in green). This result might be attributed to ADO or is possibly due to the heterogeneous mutation status in primary tumors. Considering the probability of ADO, a minimum of ten CTCs may be necessary to detect a heterozygous mutation, as previously reported [33]. 

This study had several important findings. One was that the *PIK3CA* or *KRAS* mutation was detected in CTCs, even when the corresponding primary tumor was judged to be a wild type (Pt. Nos. 8, 12, and 17 for *PIK3CA* and Pt. No.19 for *KRAS*; Table 3, highlighted in red). This finding may reflect a heterogeneous gene status for *PIK3CA* and *KRAS* in primary tumors. It is assumed that this single-cell mutational analysis method may facilitate the detection of initial gene alterations that are at a minority stage in primary tumors. Potentially, minority clones with a mutation might establish metastases. This issue should be clarified in a prospective study. Another notable finding was that our CTC capture system could detect mutations, both in CK-positive epithelial CTCs and in CK-negative CTCs, which lack epithelial features (Pt. Nos.17 and 19). The third finding was the association of CTC with lung metastasis, but not with liver metastasis. Considering that lung metastasis is established via systemic venous circulation rather than the portal vein system, this association seems to be reasonable.

However, we should emphasize that the results should be interpreted with caution due to the small number of patients included in this study. In the future, our results could be confirmed in studies that include greater numbers of patients with CRC. 

The present study identified some of the gene mutations associated with CRC. Future investigations could also include CRC-specific mutations in other genes, such as *p53, APC*, and *β-catenin*. That approach could increase the rate of detecting mutations in CTCs. In CRC, the rates of mutations are 50% in *p53* and 90% in *APC* or *β-catenin* [34]. Moreover, collecting viable CTCs could be useful in clarifying the biological characteristics of CTCs with single-cell analyses. For example, with the C1 Single-Cell Auto Prep system (Standard BioTools, K.K., Tokyo, Japan), it is possible to analyze the whole transcriptome and the whole genome at the single-cell level.

In conclusion, we demonstrated that a dielectrophoretic micropore system equipped with a micromanipulator could facilitate the detection of CTCs from patients with CRC, irrespective of epithelial marker expression. We showed that single-cell mutation analyses were feasible by targeting mutational hotspots. This system can be applied to the analysis of various cancer types; thus, this tool can contribute to accelerating CTC research.

## Figures and Tables

**Table 1:** Single cell analysis (CRC cell line).

Cell Line	Mutation	Pool	Single Cell			
			①	②	③	④
DLD-1	KRAS	**G13D(he)**	wt	wt	**G13D(he)**	**G13D**
	BRAF	wt	wt	wt	wt	wt
	PIK3CA	wt	wt	N.D	wt	wt
			①	②	③	④
RKO	KRAS	wt	wt	wt	wt	wt
	BRAF	**V600E(he)**	**V600E(he)**	**V600E(he)**	wt	wt
	PIK3CA	**H1047R(he)**	wt	**H1047R**	**H1047R(he)**	**H1047R(he)**
			①	②	③	④
HCT116	KRAS	**G13D(he)**	**G13D**	wt	**G13D**	wt
	BRAF	wt	wt	wt	wt	wt
	PIK3CA	**H1047R(he)**	wt	wt	wt	**H1047R**

N.D: not determined. V600E shows V = Val (Valine) changes to E = Glu (Glutamic acid) at the codon 800.

**Table 2:** Detection of CTC in CRC patients (n = 24).

Pt.	Location	Differentiation	TNM	Stage	Synchronous Meta. /Metachronous Meta.	Metastasis at Other Sites	CK(+) Cell Number	CK(−) Cell Number
1	Rb	muc	T3N2M0	IIIB	metachronous	Liver, Bone	0	-
2	A	tub2	T4bN2M0	IIIC	-	-	0	-
3	Ra	-	-	IV	synchronous	Liver	0	-
4	Rb	tub2	T3N2M0	IIIB	metachronous	LN	0	-
5	S	tub2	T3N0M0	IIA	metachronous	Liver, Peritoneum	3	-
6	RS	-	-	IV	synchronous	Liver, Lung, Bone	5	-
7	T	tub1	T3N1M1	IV	synchronous	Lung	3	-
8	V	tub1	T4bN1M1	IV	synchronous	Liver	4	-
9	A	tub2	T4aN1M1	IV	synchronous	Liver	0	-
10	S	tub1	T3N1M1	IV	synchronous	Liver	0	-
11	S	tub2	T2N1M0	IIIA	metachronous	Liver	0	-
12	Rb	tub2	T3N0M1	IV	synchronous	Liver	1	-
13	S	tub2	T4aN1M1	IV	synchronous	Liver	0	-
14	RS	tub2	T2N1M0	IIIA	-	-	0	-
15	A	no data	T3N1M1	IV	synchronous	Liver	1	-
16	S	tub2	T3N2M0	IIIB	metachronous	Lung, LN	0	2
17	S	tub2	T3N1M0	IIIB	metachronous	Lung	1	2
18	A	tub2	T4bN2M0	IIIC	metachronous	Liver	0	1
19	Rb	tub1	T3N0M1	IV	synchronous	Liver	0	4
20	Rb	tub1	T3N0M1	IV	synchronous	Liver	0	5
21	C	tub2	T3N1M0	IIIB	metachronous	Liver	0	2
22	A	tub1	T3N1M0	IIIB	metachronous	Liver	0	3
23	RS	tub2	T3N0M0	IIA	-	-	0	5
24	RS	tub2	T4bN2M1	IV	synchronous	Liver, Lung, Peritoneum	0	1

**meta.:** metastasis, Pt: patient, LN: lymph node.

**Table 3:** Single cell analyses of CK-positive and CK-negative CTCs.

CK	Pt. No.	Cell. No.	Chemotherapy	CTC			Primary Tumor		
				KRAS	BRAF	PIK3CA	KRAS	BRAF	PIK3CA
Positive	5	1	yes	wt	wt	wt	wt	wt	wt
		2		wt	wt	wt			
	6	1	no	wt	wt	wt	wt	wt	wt
		2		wt	wt	wt			
	7	1	no	wt	wt	wt	**G12D(he)**	wt	wt
		2		wt	wt	wt			
		3		wt	wt	wt			
	8	1	no	wt	wt	**H1047R(he)**	wt	wt	wt
		2		wt	wt	**H1047R(he)**			
		3		wt	wt	wt			
		4		wt	wt	wt			
	12	1	yes	wt	wt	**H1047R(he)**	**G12D(he)**	wt	wt
	15	1	yes	wt	wt	wt	Not available		
	17	1	no	wt	wt	wt	wt	wt	wt
Negative	16	1	yes	wt	wt	**wt**	wt	wt	wt
		2		wt	wt	wt			
	17	2	no	wt	wt	**H1047R(he)**	wt	wt	wt
		3							
				wt	wt	wt			
	18	1	no	wt	wt	wt	wt	wt	wt
	19	1	yes	wt	wt	wt	wt	wt	wt
		2		wt	wt	wt			
		3		**G13S**	wt	wt			
		4		wt	wt	wt			
	20	1	yes	wt	wt	wt	wt	wt	wt
		2		wt	wt	wt			
		3		wt	wt	wt			
		4		wt	wt	wt			
		5		wt	wt	wt			
	22	1	no	wt	wt	wt	Not available		
	23	1	no	wt	wt	wt	wt	wt	wt
		2		wt	wt	wt			
		3		wt	wt	wt			
		4		wt	wt	wt			
		5		wt	wt	wt			
	24	1	no	wt	wt	wt	wt	wt	wt

wt: wild type, G13S: G (Glycine) replaced by S (Serine) at the codon 13. H1047R: H (Histidine) replaced by R (Arginine) at the codon 1047. G12D: G (Glycine) replaced by D (Asparatic Acid) at the codon 12.

**Table 4:** Relationship between CTC and clinicopathological parameters.

	N = 24	CTC (+) N = 15	CTC (−) N = 9	*p* Value
Age: years; median (range)	67 (40–79)	69 (41–79)	59 (40–74)	0.16
Sex: Male/Female	16/8	10/5	6/3	1.00
CEA ng/mL	22 (2–17,200)	13 (2–391)	161 (3–17,200)	0.18
CA19-9 U/mL	32 (2–13,301)	31 (2–192)	53 (2–13,301)	0.16
Location: Right/Left	8/16	6/9	2/7	0.36
Max diameter: mm; median (range)	46.5 (15–90)	45 (30–90)	46.5 (15–90)	0.78
* Differentiation: tub/muc	20/1	13/0	7/1	0.16
Stage II, III/IV	12/12	7/8	5/4	0.67
Metastatic type: Metachronous/synchronous	9/12	6/8	3/4	1.00
History of chemotherapy yes/no	12/12	6/9	6/3	0.20
Liver metastasis yes/no	17/7	11/4	6/3	0.69
Lung metastasis yes/no	5/19	5/10	0/9	0.02

* tub: tubular adenocarcinoma, muc: mucinous carcinoma.

## Data Availability

Not applicable.

## Supplementary Materials

The following supporting information can be downloaded at: https://www.mdpi.com/article/10.3390/biomedicines11010203/s1, Supplementary Figure S1: Determining the cell recovery rate with the spike test. Supplementary Figure S2: Determining the melting temperature (Tm) of primers that target the point mutation regions in *KRAS*, *BRAF*, and *PIK3CA*. Supplementary Table S1: Chemotherapy regimens applied to 24 patients with CRC, prior to blood draws.

## References

[1] Siegel R.L., Miller K.D., Jemal A. (2017). Cancer statistics. CA Cancer J. Clin..

[2] Wittekind C., Neid M. (2005). Cancer invasion and metastasis. Oncology.

[3] Cremolini C., Rossini D., Dell’Aquila E., Lonardi S., Conca E., Del Re M., Busico A., Pietrantonio F., Danesi R., Aprile G. (2019). Rechallenge for Patients With RAS and BRAF Wild-Type Metastatic Colorectal Cancer With Acquired Resistance to First-line Cetuximab and Irinotecan: A Phase 2 Single-Arm Clinical Trial. JAMA Oncol..

[4] Marcuello M., Vymetalkova V., Neves R.P., Duran-Sanchon S., Vedeld H.M., Tham E., van Dalum G., Flügen G., Garcia-Barberan V., Fijneman R.J.A. (2019). Circulating biomarkers for early detection and clinical management of colorectal cancer. Mol. Asp. Med..

[5] Tarazona N., Gimeno-Valiente F., Gambardella V., Zuniga S., Rentero-Garrido P., Huerta M., Roselló S., Martinez-Ciarpaglini C., Carbonell-Asins J.A., Carrasco F. (2019). Targeted next-generation sequencing of circulating-tumor DNA for tracking minimal residual disease in localized colon cancer. Ann. Oncol..

[6] Yamada T., Matsuda A., Koizumi M., Shinji S., Takahashi G., Iwai T., Takeda K., Ueda K., Yokoyama Y., Hara K. (2019). Liquid Biopsy for the Management of Patients with Colorectal Cancer. Digestion.

[7] Tie J., Cohen J.D., Wang Y., Christie M., Simons K., Lee M., Wong R., Kosmider S., Ananda S., McKendrick J. (2019). Circulating Tumor DNA Analyses as Markers of Recurrence Risk and Benefit of Adjuvant Therapy for Stage III Colon Cancer. JAMA Oncol..

[8] Peeters D., De Laere B., Van den Eynden G., Van Laere S., Rothe F., Ignatiadis M., Sieuwerts A., Lambrechts D., Rutten A., Van Dam P. (2013). Semiautomated isolation and molecular characterisation of single or highly purified tumour cells from CellSearch enriched blood samples using dielectrophoretic cell sorting. Br. J. Cancer.

[9] Allen J.E., EI-Deiry W.S. (2010). Circulating tumor cells and colorectal cancer. Curr. Colorectal. Cancer Rep..

[10] Huang X., Gao P., Song Y., Sun J., Chen X., Zhao J., Liu J., Xu H., Wang Z. (2014). Relationship between circulating tumor cells and tumor response in colorectal cancer patients with chemotherapy; a meta-analysis. BMC Cancer.

[11] Cohen S.J.A., Punt C.J., Iannotti N., Saidman B.H., Sabbath K.D., Gabrail N.Y., Picus J., Morse M., Mitchell E., Miller M.C. (2008). Relationship of circulating tumor cells to tumor response, progression-free survival, and overall survival in patients with metastatic colorectal cancer. J. Clin. Oncol..

[12] De Bono J.S., Scher H.I., Montgomery R.B., Parker C., Miller M.C., Tissing H., Doyle G.V., Terstappen L.W., Pienta K.J., Raghavan D. (2008). Circulating tumor cells predict survival benefit from treatment in metastatic castration-resistant prostate cancer. Clin. Cancer Res..

[13] Cristofanilli M., Budd G.T., Ellis M.J., Stopeck A., Matera J., Miller M.C., Reuben J.M., Doyle G.V., Allard W.J., Terstappen L.W. (2004). Circulating tumor cells, disease progression, and survival in metastatic breast cancer. N. Engl. J. Med..

[14] Pantel K., Brakenhoff R.H., Branbt B. (2008). Detection, clinical relevance and specific biological properties of disseminating tumour cells. Nat. Rev. Cancer.

[15] Cayrefourcq L., Mazard T., Joosse S., Solassol J., Ramos J., Assenat E., Schumacher U., Costes V., Maudelonde T., Pantel K. (2015). Establishment and characterization of a cell line from human circulating colon cancer cells. Cancer Res..

[16] Gasch C., Bauernhofer T., Pichler M., Langer-Freitag S., Reeh M., Seifert A.M., Mauermann O., Izbicki J.R., Pantel K., Riethdorf S. (2013). Heterogeneity of epidermal growth factor receptor status and mutations of KRAS/PIK3CA in circulating tumor cells of patients with colorectal cancer. Clin. Chem..

[17] Morimoto A., Mogami T., Watanabe M., Iijima K., Akiyama Y., Katayama K., Futami T., Yamamoto N., Sawada T., Koizumi F. (2015). High-Density Dielectrophoretic Microwell Array for Detection, Capture, and Single-Cell Analysis of Rare Tumor Cells in Peripheral Blood. PLoS ONE.

[18] Sobin L.H., Gospodarowicz M.K., Wittekind C. (2016). UICC TNM Classification of Malignant Tumors.

[19] Fabbri F., Carloni S., Zoli W., Ulivi P., Gallerani G., Fici P., Chiadini E., Passardi A., Frassineti G.L., Ragazzini A. (2013). Detection and recovery of circulating colon cancer cells using a dielectrophoresis-based device: KRAS mutation status in pure CTCs. Cancer Lett..

[20] Gupta V., Jafferji I., Garza M., Melnikova V.O., Hasegawa D.K., Pethig R., Davis D.W. (2012). ApoStream™, a new dielectrophoretic device for antibody independent isolation and recovery of viable cancer cells from blood. Biomicrofluidics.

[21] Shim S., Stemke-Hale K., Tsimberidou A.M., Noshari J., Anderson T.E., Gascoyne P.R.C. (2013). Antibodyindependent isolation of circulating tumor cells by continuous-flow dielectrophoresis. Biomicrofluidics.

[22] Swennenhuis J.F., Reumers J., Thys K., Aerssens J., Terstappen L.W. (2013). Efficiency of whole genome amplification of single circulating tumor cells enriched by CellSearch and sorted by FACS. Genome.

[23] Satelli A., Mitra A., Brownlee Z., Xia X., Bellister S., Overman M.J., Kopetz S., Ellis L.M., Meng Q.H., Li S. (2015). Epithelial-Mesenchymal Transitioned Circulating Tumor Cells Capture for Detecting Tumor Progression. Clin. Cancer Res..

[24] Kondo Y., Hayashi K., Kawakami K., Miwa Y., Hayashi H., Yamamoto M. (2017). KRAS mutation analysis of single circulating tumor cells from patients with metastatic colorectal cancer. BMC Cancer.

[25] Kidess-Sigal E., Liu H.E., Triboulet M.M., Che J., Ramani V.C., Visser B.C., Poultsides G.A., Longacre T.A., Marziali A., Vysotskaia V. (2016). Enumeration and targeted analysis of KRAS, BRAF and PIK3CA mutations in CTCs captured by a label-free platform: Comparison to ctDNA and tissue in metastatic colorectal cancer. Oncotarget.

[26] Noura S., Yamamoto H., Miyake Y., Kim B.N., Takayama O., Seshimo I., Ikenaga M., Ikeda M., Sekimoto M., Matsuura N. (2002). Immunohistochemical assessment of localization and frequency of micrometastases in lymph nodes of colorectal cancer. Clin. Cancer Res..

[27] Yokobori T., Iinuma H., Shimamura T., Imoto S., Sugimachi K., Ishii H., Iwatsuki M., Ota D., Ohkuma M., Iwaya T. (2013). Plastin3 is novel marker for circulating tumor cells undergoing the epithelial-mesenchymal transition and is associated with colorectal cancer prognosis. Cancer Res..

[28] Alix-Panabieres C., Pantel K. (2014). Challenges in circulating tumour cell research. Nat. Rev. Cancer.

[29] Mani S.A., Guo W., Liao M.-J., Eaton E.N., Ayyanan A., Zhou A.Y., Brooks M., Reinhard F., Zhang C.C., Shipitsin M. (2008). The epithelial-mesenchymal transition generates cells with properties of stem cells. Cell.

[30] Tam W.L., Weinberg R.A. (2013). The epigenetics of epithelial-mesenchymal plasticity in cancer. Nat. Med..

[31] Watanabe T., Yoshino T., Uetake H., Yamazaki K., Ishiguro M., Kurokawa T., Saijo N., Ohashi Y., Sugihara K. (2013). KRAS mutational status in Japanese patients with colorectal cancer: Results from a nationwide, multicenter, cross-sectional study. Jpn. J. Clin. Oncol..

[32] Qin B.-D., Jiao X.-D., Liu K., Wu Y., He X., Liu J., Qin W.-X., Wang Z., Zang Y.-S. (2019). Basket Trials for Intractable Cancer. Front. Oncol..

[33] Colin M.C., Jacob S.A., Shonan S., Shuang H., Qingyu L., Carolyn H., Min S., Xinfang L., Matthew M.R., Zev A.W. (2016). Reality of Single Circulating Tumor Cell Sequencing for Molecular Diagnostics in Pancreatic Cancer. J. Mol. Diagn..

[34] Sjöblom T., Jones S., Wood L.D., Parsons D.W., Lin J., Barber T.D., Mandelker D., Leary R.J., Ptak J., Silliman N. (2006). The consensus coding sequences of human breast and colorectal cancers. Science.

