# Supplementary material for: Single-Cell Analysis of Circulating Tumor Cells from Patients with Colorectal Cancer Captured with a Dielectrophoresis-Based Micropore System
Source: Biomedicines. 2023 Jan 13;11(1):203. doi: 10.3390/biomedicines11010203 (PMC9855761; doi:10.3390/biomedicines11010203)
Supplement: Supplementary file 1 [file biomedicines-11-00203-s001.zip › biomedicines-2108658-supplementary.pdf]

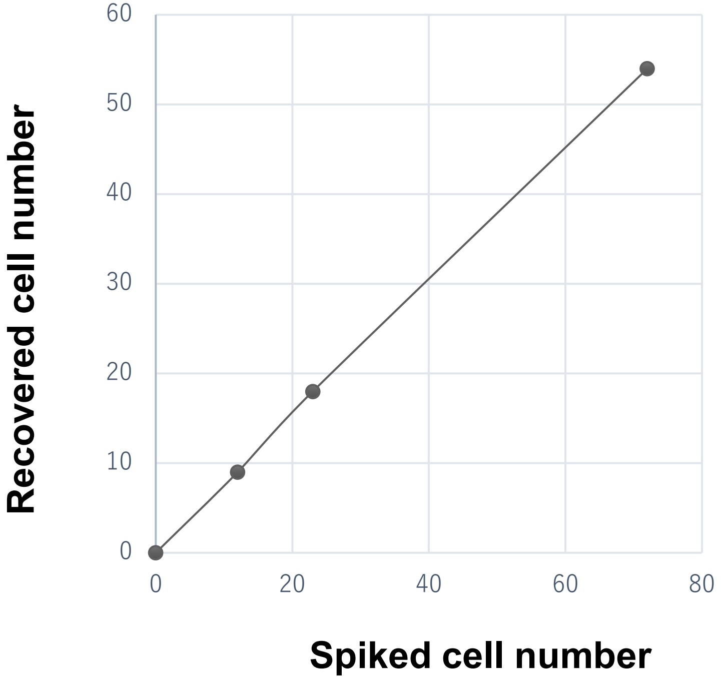

No. Cells spiked	No. Cells recovered	Recovery rate (%)
0	0	-
12	9	75
23	18	78.2
72	54	75

Supplementary Figure S1. Determining the cell recovery rate with the spike test. A suspension of cells from the colon cancer cell line, HT29, was injected into the micropore chamber. The recovery rate is the number recovered divided by the number injected (×100%). For these cells, the recovery rate was about 75%.

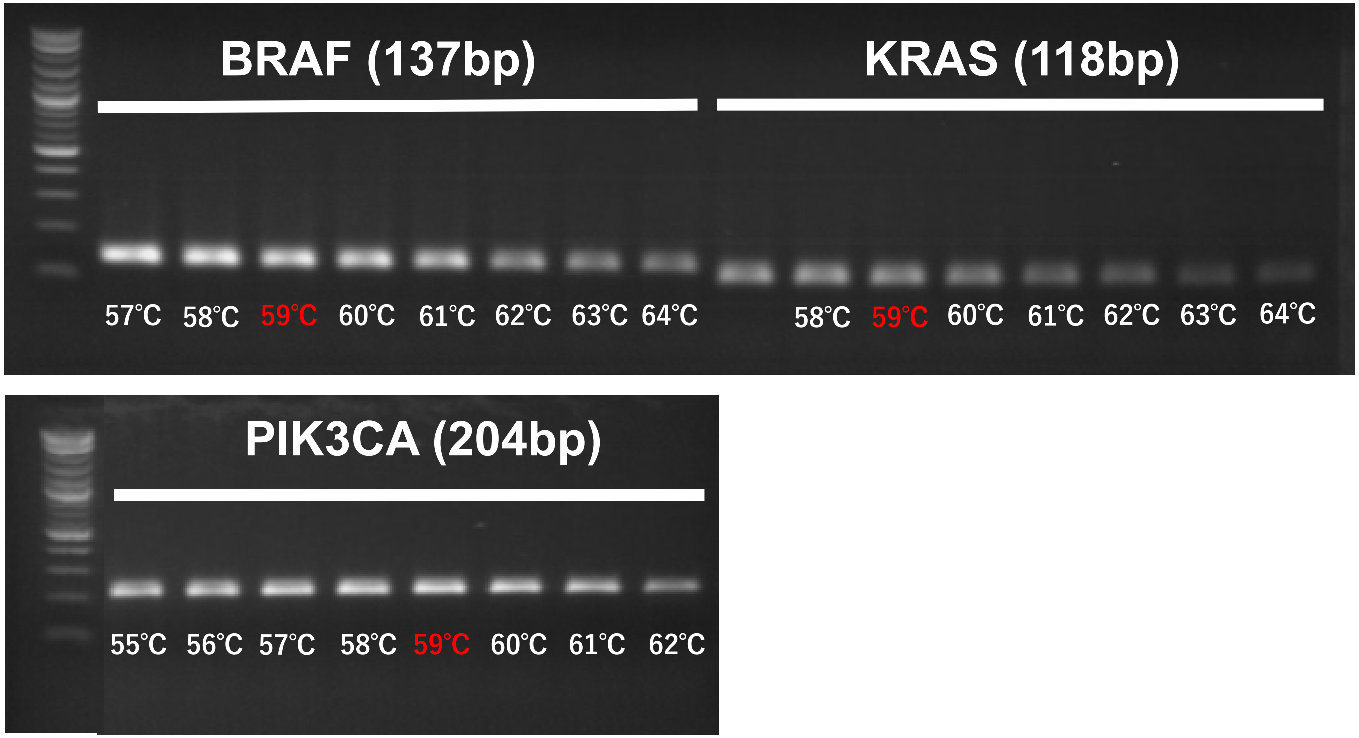

Supplementary Figure S2. Determining the melting temperature (Tm) of primers that target the point mutation regions in *KRAS*, *BRAF*, and *PIK3CA*. DNA amplification was performed with the polymerase chain reaction. Tm values were tested from 57 °C to 64 °C for each primer, with RNA extracted from CRC cell lines. The results of cells from the RKO cell line are shown here.

Table S1. Chemotherapy regimens applied to 24 patients with CRC, prior to blood draws.

Pt. No	chemotherapy
1	Capecitabine
2	-
3	-
4	XELOX+Bev
5	SOX+Bev, XELOX+Bev, CPT11+Pmab
6	-
7	-
8	-
9	XELOX+Bev
10	UFT/LV
11	XELOX
12	XELOX
13	FOLFOX
14	-
15	XELOX+Bev, FOLFOX+Bev, FOLFIRI+Bev
16	XELOX+Bev
17	-
18	-
19	XELOX+Bev
20	XELOX+Bev
21	-
22	-
23	-
24	-
